# Effects of seat surface inclination on respiration and speech production in children with spastic cerebral palsy

**DOI:** 10.1186/s40101-015-0057-3

**Published:** 2015-04-24

**Authors:** Hwa-Kyung Shin, Eun-Jin Byeon, Seok Hun Kim

**Affiliations:** Department of Physical therapy, Catholic University of Daegu, 13-13 Hayang-ro, Hayang-eup, Gyeongsan 712-702 Korea; School of Physical Therapy & Rehabilitation Sciences, University of South Florida, 12901 Bruce B. Downs Blvd., MDC 77, Tampa, FL 33612 USA

**Keywords:** Cerebral palsy, Ergonomics, Respiration, Seat inclination, Speech production

## Abstract

**Background:**

Respiratory and speech problems are commonly observed in children with cerebral palsy (CP). The purpose of this study was to identify if inclination of seat surface could influence respiratory ability and speech production in children with spastic diplegic CP.

**Methods:**

Sixteen children with spastic diplegic CP, ages 6 to 12 years old, participated in this study. The subjects’ respiratory ability (forced vital capacity (FVC), forced expiratory volume in 1 s (FEV1), peak expiratory flow (PEF), and maximum phonation time (MPT)) were measured in three sitting conditions: a seat surface inclined 0°, anterior 15°, and posterior 15°.

**Results:**

FVC was significantly different across three inclinations of seat surface, *F*(2, 45) = 3.81, *P* = 0.03. In particular, the subjects’ FVC at a seat surface inclined anterior 15° was significantly greater than at a seat surface inclined posterior 15° (*P* < 0.05). However, FEV1, PEF, and MPT were not significantly affected by seat surface inclination (*P* > 0.05).

**Conclusions:**

The results suggest that anterior inclination of seat surface may provide a positive effect on respiratory function in children with spastic diplegic CP.

## Background

Children with spastic cerebral palsy (CP) account for about 80% of all children with CP. They frequently show abnormal muscle tone, impaired muscle contraction, and altered postural control [1]. These abnormal muscle tones and movements not only adversely affect development of the trunk muscles that are the foundation of respiration, but also cause thoracic deformity, which leads to respiratory problems [2].

Approximately 40% of children with spastic CP experience the impairments of respiratory function [3]. The level of respiratory impairment is significantly greater in children with spastic diplegic CP than in those with spastic Hemiplegic CP [4]. Various therapeutic approaches have been introduced to improve respiratory functions in children with CP, such as strengthening respiratory muscles [5], inhibiting muscle tone [5], swimming [6], treadmill walking [7], and cycle ergometer training [8].

An ergonomic approach that adjusts the orientation of sitting to support physical function has been studied in children with CP [9-13]. Evidence shows that the orientation of sitting position influences functions in children with CP [14], especially those who depend on a wheelchair for most of their daily activities [15]. The anterior-inclined seat may provide a positive effect to the function of the upper limbs [15] and postural control [14,16]. Studies have also reported that the inclination of sitting position affects the respiratory function and speech in children with CP [12,17]. Nevertheless, the effectiveness of the sitting position on these functions is inconclusive [14,18]. Thus, the purpose of this study was to investigate whether an ergonomic approach using three levels of seat inclination could affect respiratory patterns and maximum phonation in children with spastic diplegic CP.

## Methods

### Subjects

Sixteen children with spastic diplegic CP were recruited from the Gumi area in Korea for this study (Table 1). Inclusion criteria were as follows: children who have a) an ability to maintain a sitting position independently, b) the Gross Motor Function Classification System (GMFCS) levels I to IV [19], and c) an ability to follow the examiner’s instruction. Subjects who had a) any neurological disorder other than cerebral palsy and b) any orthopedic problem that limits the sitting balance were excluded from this study. This study was conducted in compliance with the ethical standards of the Declaration of Helsinki, and a parent or guardian of each child signed a written informed consent form prior to the experiment.

**Table 1:** **Clinical characteristics of the children with cerebral palsy**

**Variables**	**Mean ± SD** ^**a**^	**Range**
Gender (M/F)	8/8	
Age (year)	10.06 ± 1.98	6 ~ 12
Height (cm)	127.31 ± 8.97	105 ~ 139
Weight (kg)	28.36 ± 6.44	18 ~ 42
GMFCS level	2.44 ± 1.21	I ~ IV

^a^All values represent mean ± SD except gender. GMFCS, Gross Motor Function Classification System.

### Experimental procedures and equipment

All experiments were performed at a local rehabilitation center. Each subject participated in one study session that took approximately 1 h to complete. The subjects were closely guarded by a physical therapist throughout the experiment.

#### Sitting positions on the inclination chair

**Figure 1:**
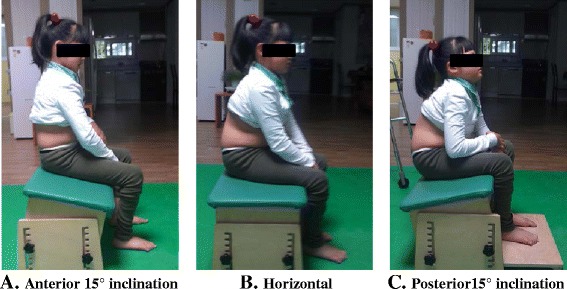
Three positions of seat surface inclination**.** Subjects’ respiratory function and speech production were assessed in three seat inclination conditions: **(A)** 15° of anterior inclination, **(B)** 0° (horizontal) of inclination, and **(C)** 15° of posterior inclination.

The chair used in this study was an adjustable bench-style chair without a backrest. The subjects were asked to sit on the chair with its seat surface inclined anterior 15°, horizontal (0°), and posterior 15° [20] (Figure 1), and the order of sitting conditions was randomly selected for assessments of respiratory function and maximum phonation time. At each sitting, their feet touched the floor maintaining 90° of their knee joint angle, and when needed, a foothold or a footrest was used to secure their feet to the floor [21] (Figure 1C). For each sitting position, the subjects were given a 5-min adaptation period to get used to the change so that their respiration would be regular prior to the first testing trial. If needed, the subjects’ parents or physical therapists provided them with minimal assistance to maintain their postures.

#### Measurement of respiratory function

The Cardio Touch 3000S (Bionet Co., Seoul, Korea) was used to measure respiratory function including forced vital capacity (FVC), forced expiratory volume in 1 s (FEV1), and peak expiratory flow (PEF). Subjects wore comfortable pants to minimize abdominal compression which might interfere with their respiratory activities during the assessments. FVC was determined by measuring the amount of a quick and strong expiration after a maximal inspiration. FEV1 was defined as the expiration amount of air for 1 s following a maximal inspiration, and PEF was determined by calculating the speed of the air emitted maximally. To ensure the subjects’ understanding of the experiment, appropriate instructions and demonstrations were provided prior to the assessments. Each subject’s nose was blocked to prevent air leakage. The subjects had a total of three trials with at least 3-min rest between trials, and the maximum value was used for further analysis.

#### Measurement of speech production

To assess speech production, the maximum phonation time (MPT) was determined using a stopwatch to measure the duration of the pronunciation of a vowel sound (‘ah’). To enhance the credibility of the result, a speech therapist recorded and evaluated the phonation. The subjects performed a total of three trials for each randomly selected sitting position (seat surface inclined anterior 15°, 0°, or posterior 15°) at their most comfortable pitch and strength, and the maximum value was used for further analysis.

### Statistical analysis

One-way ANOVA was used to assess differences in FVC, FEV1, PEF, and MPT among three inclinations of seat surface using the IBM SPSS Statistics Software 22.0 (IBM Co., Armonk, NY, USA). Significance level was set at *P* < 0.05 for each variable. When a significant difference was found, a *post hoc* analysis using Turkey tests was conducted to determine where the difference existed.

## Results

### Respiration

**Figure 2:**
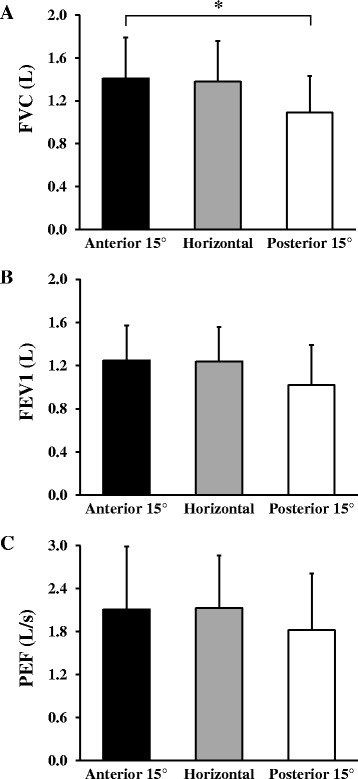
Comparison of respiration function in three positions of seat surface inclination. The asterisk indicates a significant difference between 15° of anterior inclination and 15° of posterior inclination (*P* < 0.05). **(A)** FVC, **(B)** FEV1, and **(C)** PEF represent forced vital capacity (liter (L)), forced expiratory volume in 1 s (L), and peak expiratory flow (L/second (L/s)). Error bars represent standard deviations (SD).

FVC was significantly different across three seat inclination positions, *F*(2, 45) = 3.81, *P* = 0.03. *Post hoc* analysis indicated that FVC at anterior 15° inclination of seat surface (1.41 ± 0.38 L) was significantly greater than at posterior 15° inclination of seat surface (1.38 ± 0.38 L) (*P* < 0.05, Figure 2), but not significantly different from that of horizontal inclination (1.09 ± 0.34 L) (*P* > 0.05). No significant differences were found in FEV1, *F*(2, 45) = 2.34, *P* = 0.11, and PEF, *F*(2, 45) = 1.27, *P* = 0.21 across the three seat surface inclinations. Nonetheless, FEV1 and PEF at anterior 15° (1.25 ± 0.32 L and 2.11 ± 0.88 L/s, respectively) and horizontal inclinations (1.24 ± 0.32 L and 2.13 ± 0.73 L/s, respectively) tended to be greater than those at posterior 15° inclination (1.02 ± 0.37 L and 1.82 ± 0.79 L/s, respectively).

### Speech production

**Figure 3:**
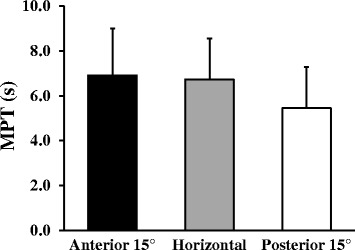
Comparison of maximum phonation time for speech production in three positions of seat surface inclination. MPT represents maximum phonation time (s). Error bars represent SD.

MPT of pronunciation of a vowel sound (‘ah’) was greater at a seat surface inclined anterior 15° (6.91 ± 2.09 s) than when inclined 0° (6.73 ± 1.81 s) and posterior 15° (5.45 ± 1.83 s). However, no significant difference was found in MPT across the three seat surface inclinations, *F*(2, 45) = 2.80, *P* = 0.07 (Figure 3).

## Discussion

Children with CP experience various pulmonary problems [22]. In particular, respiratory function is markedly diminished in children with spastic diplegic CP, which may be due to the inefficient functions of respiratory muscles [4]. Altered breathing patterns, such as unsynchronized movements between the diaphragm and respiratory muscles, may result in shallow and rapid respiration in children with spastic CP [23]. Impairments in postural control may also contribute to abnormal respiratory function in children with spastic CP [20]. Studies have shown that the vital capacity varies depending on posture (for example, sitting position at various seat inclinations) [20] and this variation was significant in children with spastic diplegic CP [24].

An ergonomic approach has been recognized as a simple intervention to improve respiratory function in children with CP. A sitting device such as a wheelchair can be modified according to individual needs by inserting a frame and attaching a foothold or a backrest [16,25]. The modification of sitting posture changes the structure of respiratory muscles in resting position. Change in sitting posture can enhance the respiratory muscle activities, reduce the risk of airway blockage, and ultimately improve respiratory functions [25,26]. The chair-inclination intervention is an ergonomic approach that can affect posture and also can change function of the respiratory system. Respiration and speech production in healthy adults are affected by the chair tilt that helps maintain the neutral posture of the head and the trunk and promotes stable activities of the respiratory muscles [27,28]. In children with CP, an appropriate hip joint angle is important for stable and balanced sitting, and the chair surface, which is used as a means of adjusting the hip joint angle, affects the change in spatial adjustment and therefore safety for the user [14-16,20].

A decrease in FVC is a major indicator of impaired respiratory function in children with spastic diplegic CP [4]. This present study shows that the FVC of children with spastic diplegic CP was greater with 15° of anterior seat inclination than with 15° of posterior seat inclination. The result suggests that sitting in an anterior-inclined seat may help children with CP maintain or improve their respiratory function. Children with spastic CP, because of their abnormal muscle tone and unstable posture, often display a particular way of sitting. To compensate for their unstable posture, their sitting position involves upper trunk flexion and posterior pelvic tilt [26]. Studies demonstrated that the anterior seat inclination helped the trunk extend into a more upright position [29,30]. The adapted sitting posture may allow the inspiratory and expiratory muscles to function more efficiently (for example, increased FVC). Whereas, the posterior seat inclination may increase both upper trunk flexion and posterior pelvic tilt, thereby adversely affecting the respiratory function.

Our study did not show a significant difference in either FEV1 or PEF over the three sitting positions, although the variables at anterior 15° and horizontal inclinations were notably greater than those at posterior 15° inclination. The result may be because FEV1 and PEF are not sensitive enough to identify the changes in respiratory function across the three seat inclinations. Moreover, the differences of respiratory measures were minimal between the anterior 15° and horizontal inclinations, which were consistent with a previous study [31]. A long-term intervention study may provide better insight into the effects of anterior seat inclination on respiratory function in children with CP. In addition, sitting on an anterior-inclined seat for extended periods of time may cause potential problems, such as excessive lumbar lordosis or lower back pain. Thus, this intervention approach should be combined with periodic assessment of sitting posture.

The MPT is often used to evaluate respiration and speech production [32]. In this study, no significant difference was observed in MPT across the three seat inclination positions. However, the results showed that children with spastic diplegic CP sitting at 15° of anterior inclination had a slightly longer MPT than sitting at the horizontal or 15° of posterior inclination position. The abnormal respiratory development may cause altered antagonistic functions of the abdominal and thoracic muscles during the expiration period. As a result, expiration is shortened, and the air flow required for speech is insufficiently formed, temporarily pausing respiration and speech production, and making it difficult to adjust loudness and pitch of the voice [33]. This respiratory pattern may affect phonation in children with spastic CP possibly because the deterioration of the abdominal muscle decreases the expiration volume and therefore phonation capacity [27]. Expiratory muscle strengthening exercise may be effective in achieving stable respiratory cycles and expiration volumes in order to eventually improve phonation [34].

Respiration is closely associated with several facets of speech sounds. The quality, duration, loudness, and pitch of phonation are greatly influenced by the condition of respiratory function. A study in people with multiple sclerosis suggested that to enhance the phonation quality, respiratory training approaches such as expiratory muscle strengthening exercise need to be combined with speech therapy [34]. Therefore, the seat-inclination intervention in daily activities or during speech therapy may accelerate positive results.

A limitation of this study was that the subjects fell within a wide range (from levels I to IV) of the GMFCS level and this may limit positive outcomes. Thus, the effect of a seat inclination approach on respiratory function and speech production in children with different levels of GMFCS needs to be addressed in future studies. It is also possible that ergonomic approaches tested in various environments and including postures other than sitting may provide children with spastic diplegic CP with more effective methods of respiratory function.

## Conclusions

In this study, the FVC, FEV1, PEF, and MPT were assessed to determine the optimal chair inclination for effective respiration and speech production of children with spastic diplegic CP. A significant difference was observed in the FVC across three seat inclination conditions. The FVC was significantly enhanced with the anterior seat inclination compared to the posterior seat inclination. These results suggest that adjustment of seat inclination may be an effective intervention to improve the respiratory function of children with spastic diplegic CP.

## Abbreviations

ANOVA: analysis of variance
CP: cerebral palsy
FEV1: forced expiratory volume in 1 s
FVC: forced vital capacity
GMFCS: Gross Motor Function Classification System
L: Liter
L/s: liter/second
M/F: male/female
MPT: maximum phonation time
PEF: peak expiratory flow
SPSS: Statistical Package for the Social Sciences.
